# Tc-Glutathione Complex (Tc -GSH) : Labelling, Chemical Characterization and Biodistribution in Rats

**DOI:** 10.1155/MBD.1999.329

**Published:** 1999

**Authors:** K. Baba, J. L. Moretti, P. Weinmann, R. Senekowitsch-Schmidtke, M. T. Ercan

**Affiliations:** 1 Université Paris Nord Laboratoire de Biophysique Expérimentale et Radiopharmacologie UPRES 2360, UFR Santé Médecine Biologie Humaine 74, rue M. Cachin Bobigny cedex 93018 France; 2 Nuklearmedizinische Klinik und Poliklinik Ismaninger Strabe 22 München D-81675 Germany; 3 Hacettepe University Medical Center Ankara Turkey

## Abstract

The chemical structure of ^99m^Tc-GSH has been estabilished using the ^99^Tc isotope. Labeling of glutathione with technetium in the presence of stanous chloride gave a high yield result. In a comparative study between ^99^Tc and ^99^Tc glutathione, the Tc-GSH complex obtained was purified and characterized by uv, visible spectroscopy, HPLC, Biogel chromatography, mass and NMR spectroscopy. Stoichiometric analysis showed a 2 : 1 molar ratio of GSH/Tc for the reaction. The molecular mass assessed by mass spectroscopy was 727 Da corresponding to an oxo(bis) glutathione technetate. NMR studies demonstrated that each glutathione molecule was coordinated to technetium via cysteinyl sulfur and nitrogen atoms. The biodistribution of the complex was studied in normal rats. Blood clearance was rapid during the first hour involving a biexponential curve ( t1/2 (1) : 50 min, t1/2 (2) : 400 min ). No radioactive accumulation was found in any specific organ except kidney and bladder. All the activity excreted was found unchanged in urine. In conclusion, Tc-GSH displayed an anionic dimer form as GSH-Tc-GSH. We assume that the complex is a tetradentate (2N,2S) complex containing a pentavalent technetium coordinated by two thiol and nitrogen atoms of both GSH ligands, and an apical oxo group.


### Tc-GLUTATHIONE COMPLEX (Tc -GSH) : LABELLING, CHEMICAL CHARACTERIZATION AND BIODISTRIBUTION IN RATS

K. Baba*[1], J. L. Moretti[1], P. Weinmann[1],

R. Senekowitsch-Schmidtke[2] and M. T. Ercan[3]

[1] Université Paris Nord, Laboratoire de Biophysique Expérimentale et Radiopharmacologie, UPRES 2360, UFR Santé Médecine Biologie Humaine, 74, rue M. Cachin, 93018 Bobigny cedex, France
[2] Nuklearmedizinische Klinik und Poliklinik, Ismaninger Strabe 22, D-81675 München, Germany
[3] Hacettepe University, Medical Center, Ankara, Turkey

**ABSTRACT.** The chemical structure of $^{99m}$Tc-GSH has been estabilished using the $^{99}$Tc isotope. Labeling of glutathione with technetium in the presence of stanous chloride gave a high yield result. In a comparative study between $^{99}$Tc and $^{99m}$Tc glutathione, the Tc-GSH complex obtained was purified and characterized by uv, visible spectroscopy, HPLC, Biogel chromatography, mass and NMR spectroscopy. Stoichiometric analysis showed a 2 : 1 molar ratio of GSH/Tc for the reaction. The molecular mass assessed by mass spectroscopy was 727 Da corresponding to an oxo(bis) glutathione technetate. NMR studies demonstrated that each glutathione molecule was coordinated to technetium via cysteinyl sulfur and nitrogen atoms. The biodistribution of the complex was studied in normal rats. Blood clearance was rapid during the first hour involving a biexponential curve ( $t_{1/2}$ (1) : 50 min , $t_{1/2}$ (2) : 400 min ). No radioactive accumulation was found in any specific organ except kidney and bladder. All the activity excreted was found unchanged in urine. In conclusion, Tc-GSH displayed an anionic dimer form as GSH-Tc-GSH. We assume that the complex is a tetradentate (2N,2S) complex containing a pentavalent technetium coordinated by two thiol and nitrogen atoms of both GSH ligands, and an apical oxo group.

### INTRODUCTION

During the recent years increasing fundamental research about the structure of Tc radiopharmaceuticals has been done using the long-lived $^{99}$Tc isotope ($t_{1/2}$= 2,13.$10^5$ yr). When using $^{99}$Tc, one must bear in mind that the complex formations of $^{99}$Tc and $^{99m}$Tc may be different, resulting from the different concentration ranges used for the two isotopes(1,2) Several investigators (3-7) have used $^{99m}$Tc-labeled sulfhydryl containing aminoacids, carbonic acids and heterocyclic compounds as a new approach in radiopharmaceutical design. It was demonstrated that small molecular weight complexes of $^{99m}$Tc with fast renal clearance or biliary excretion and no significant uptake by any other organs showed sufficient accumulation in tumors for early scintigraphic visualization (8-10). The renal route of excretion is an advantage over the biliary one, especially for the identification of abdominal lesions (9,11). This point was also emphasized by Fischman(11) with the use of chemotactic peptides. Peptides of low molecular weight labeled with $^{99m}$Tc could be a better alternative because of their faster blood clearance and excretion mainly via the kidney. Detailed structural information concerning Tc-radiopharmaceuticals is now available for Tc-AHBDP (12), Tc-glucoheptonate (13) and oxotechnetium compounds (14). Glutathione γ-glutamyl cysteinyl glycine was chosen as a model compound because of the critical fonctions it carried in humans (15). Due to its a small molecular weight, it was expected to better penetrate injured capillaries in inflammatory lesions (16), breast cancer (17), head and neck tumors(18).
More detailed structural information about the radiopharmaceuticals in use today would greatly facilitate the prediction of the in vivo stability and target-organ distribution. For this reason the study of the Tc-GSH chemical structure and its biodistribution in rats were undertaken.

### MATERIAL AND METHODS

Glutathione (reduced form), $SnCl_2$, and buffer products were purchased from Sigma Aldrich. $Na^{99m}TcO_4$ was eluted from a commercial $^{99}$Mo/$^{99m}$Tc generator (Cis BioInternational). $NH_4{}^{99}TcO_4$ was obtained from Dupont-NEN laboratories.
Labeling reaction of the Tc-GSH complex was performed with 20 mg of glutathione dissolved in 2 ml phosphate buffer while stirring and 0,3 ml of $SnCl_2.2H_2O$ (1 mg/ml of HCl 0,05N) was added. The mixture was stirred for a few minutes and filtered. 2 ml of pertechnetate were added and the mixture was shaken for few seconds and left to react for 15 min at room temperature.

The radiochemical purity of the Tc-GSH complex was assessed by ITLC using a CHROMELUC Nu-102 radiochromatogram (Numelec) with silica-gel plates and two solvents : Butanone and NaCl .

Electrophoretic experiments of the Tc-GSH complex were performed on cellulose acetate strips in phosphate buffer (0.05M, pH 7). 5 µl of each solution were placed at midpoint of the band and 200V was applied for 1h at room temperature. After drying, each strip was cut into equal lenght and the activity counted with a ß counter (Beckman, LS 6000SC) for the $^{99}$Tc-GSH complex and $\gamma$ counter (Wallac, LKB1261).

Ultraviolet and visible electron spectra of the Tc-GSH complex were recorded between 190 and 850 nm on a HEWLETT PACKARD 8452 spectrophotometer using closed 10-mm Supracil quartz cuvettes.

Infrared spectra of the Tc-GSH complex were recorded from 600-4000 $cm^{-1}$. Spectra were performed on solid samples prepared as KBr pellets.

An analytical study of the $^{99m}$Tc-GSH complex was determined by gradient HPLC (C-18 reversed phase uptisphere 5µm UP50DB-25K, Interchim) using a flow rate of 1 ml/mn equiped with a UV-Vis detector and gamma-counting system (NaI crystal ) coupled to two channel analysers and integrated by an Ezchrom software program (Merck). The complex (20µl) was eluted from the HPLC column with a gradient medium of (A) 0,05M phosphate buffer pH = 7 and (B) 30% ethanol in (A).

Gel chromatography of the Tc-GSH complex was performed on biogel P2 columns (Biorad) with the technique introduced by Persson (19). The biogel P2 was used to separate and identify smaller molecular weight compounds. The various samples were eluted with PBS (pH = 7) and the column was calibrated for different molecular weight ranges.

NMR analysis of the GSH and the Tc-GSH complex were obtained at 20°C with a Varian NMR spectrometer at 500MHz.

Fast atom bombardement mass spectroscopy (FAB$^+$ - MS) of the Tc-GSH complex were recorded with a MAT 95 spectrometer by mixing 1 µl sample with 5 µl glycerol and bombardement with caesium atoms of low energy of about 150 eV at room temperature.

Blood clearance of $^{99m}$Tc-GSH complex was studied in male sprague dawley rats (150-200g). 100 µl of $^{99m}$Tc-GSH were injected i.v. and blood samples drawn from a femoral vein at 5 min, 2h, 4h, 6h and 24h. The samples were counted in a gamma-counter. The means of percentage injected dose were plotted with the course of time.

The in vivo distribution studies were performed in male sprague dawley rats (150-200g) by injecting into the femoral vein 100 ml of $^{99m}$Tc-GSH. Five rats were studied for each data point. The rats were killed and dissected at 1h, 6h postinjection. All the organs were removed, samples of blood and urine were also obtained. The organ samples were weighted and counted in a gamma-counter ( Wallac, LKB1261)).

### RESULTS

The Tc-GSH complex displayed a negative charge on paper electrophoresis. Instant thin layer chromatography analysis results are summarized in table I. In general, free pertechnetate moved to the solvent front whereas reduced $^{99m}$Tc and bound $^{99m}$Tc-R (R-Tc) remained at the spot of origin in the butanone solvent. In the saline solvent, reduced $^{99m}$Tc stayed at the origin whereas the free pertechnetate and R-Tc moved with the solvent front. The labeling yield of $^{99m}$Tc-GSH detected by paper and thin layer chromatography was over 95%.The UV visible spectra of Tc-GSH complex showed an absorbance band at 385 nm (fig.1). Glutathione alone and glutathione-Sn did not absorb at 385 nm. The $^{99}$Tc-GSH complex was stable for more one week at room temperature. The stoechiometry for the reaction of Tc with glutathione determined at partial molar ratios at different glutathione concentrations (Sn/Tc/GSH = 1/1/X, X = 0-10) indicated the existence of two GSH coordinated with one Tc ion (fig. 2).

**Table I** : Thin layer chromatography of $^{99}$Tc and $^{99m}$Tc-GSH complex

| Compound | rf (NaCl) | rf (Butanone) |
|---|---|---|
| $^{99m}TcO_4^-$ | 1 | 1 |
| $^{99}TcO_4^-$ | 1 | 1 |
| $^{99m}$Tc-GSH | 1 | 0 |
| $^{99}$Tc-GSH | 1 | 0 |

The IR spectrum of the Tc-GSH complex showed the absence of the 2540 $cm^{-1}$ band and the presence of the vibration band of the TC=O core (931 $cm^{-1}$).

**Figure 1** : UV-Vis spectra of various compounds at 25°C

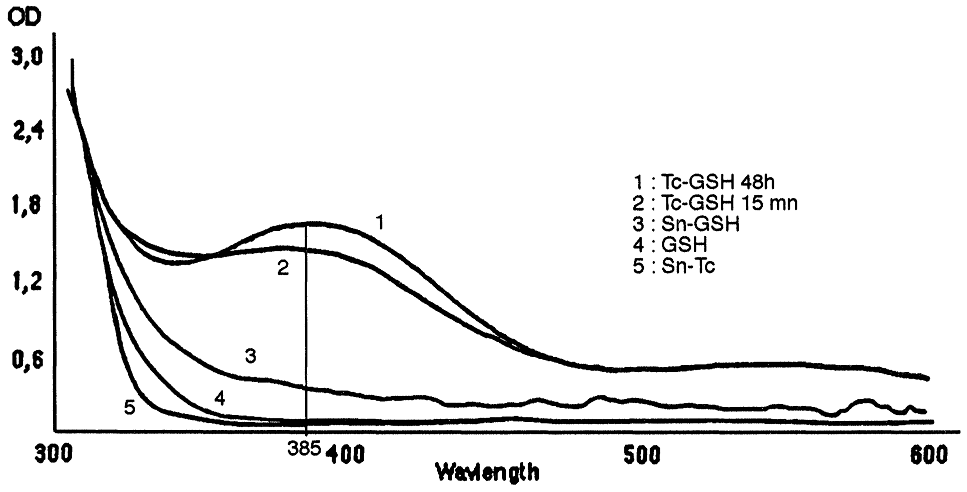

**Figure 2** : Mol-ratio reaction curve of technetium with gluthatione at 385 nm

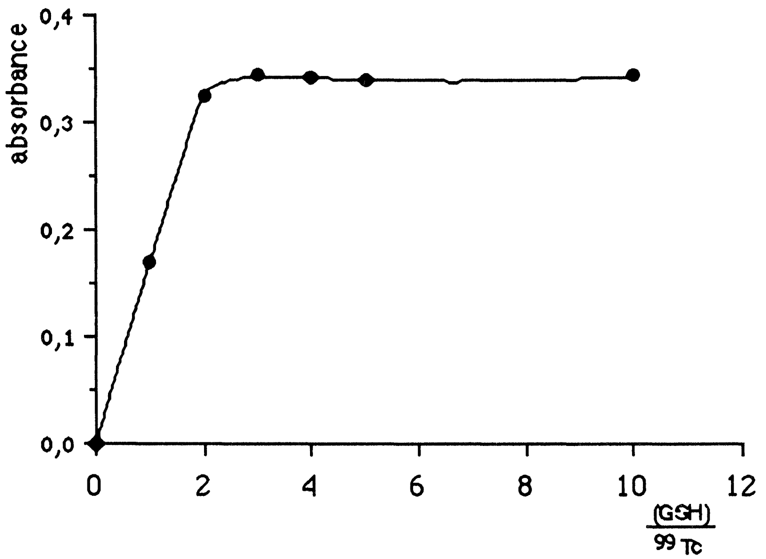

**Table II** : Proton NMR spectroscopic data of GSH and $^{99}$Tc-GSH complex

| Compound | cys δ($C_\alpha H_2$) | cys δ($C_\beta H_2$) | gly δ($C_\alpha H_2$) | glu δ($C_\alpha H_2$) | glu δ($C_\beta H_2$) | glu δ($C_\gamma H_2$) |
|---|---|---|---|---|---|---|
| GSH | 3,794 | 2,917 | 3,943 | 4,539 | 2,122 | 2,507 |
| | 3,807 | 2,929 | 3,953 | 4,550 | 2,137 | 2,521 |
| | 3,820 | 2,936 | | | 2,150 | 2,537 |
| | | | | | 2,165 | 2,552 |
| Tc-GSH | 3,767 | 2,956 | 3,791 | 4,550 | 2,158 | 2,558 |
| | 3,779 | 2,971 | 3,813 | 4,563 | 2,170 | 2,573 |
| | | | | | 2,185 | 2,583 |
| δ: ppm | | | | | 2,199 | 2,597 |

The NMR spectrum of the GSH and the Tc-GSH complex demonstrated the disappearance of fine structure of the signal (fig. 3). The proton signal (table II) indicated, in particular, that protons bound to cysteinyl α and β carbons appeared to be altered.

**Figure 3** : $^{1}H$ NMR spectra of GSH (A) and $^{99}Tc$-GSH complex (B) at 500 MHz, 20°C

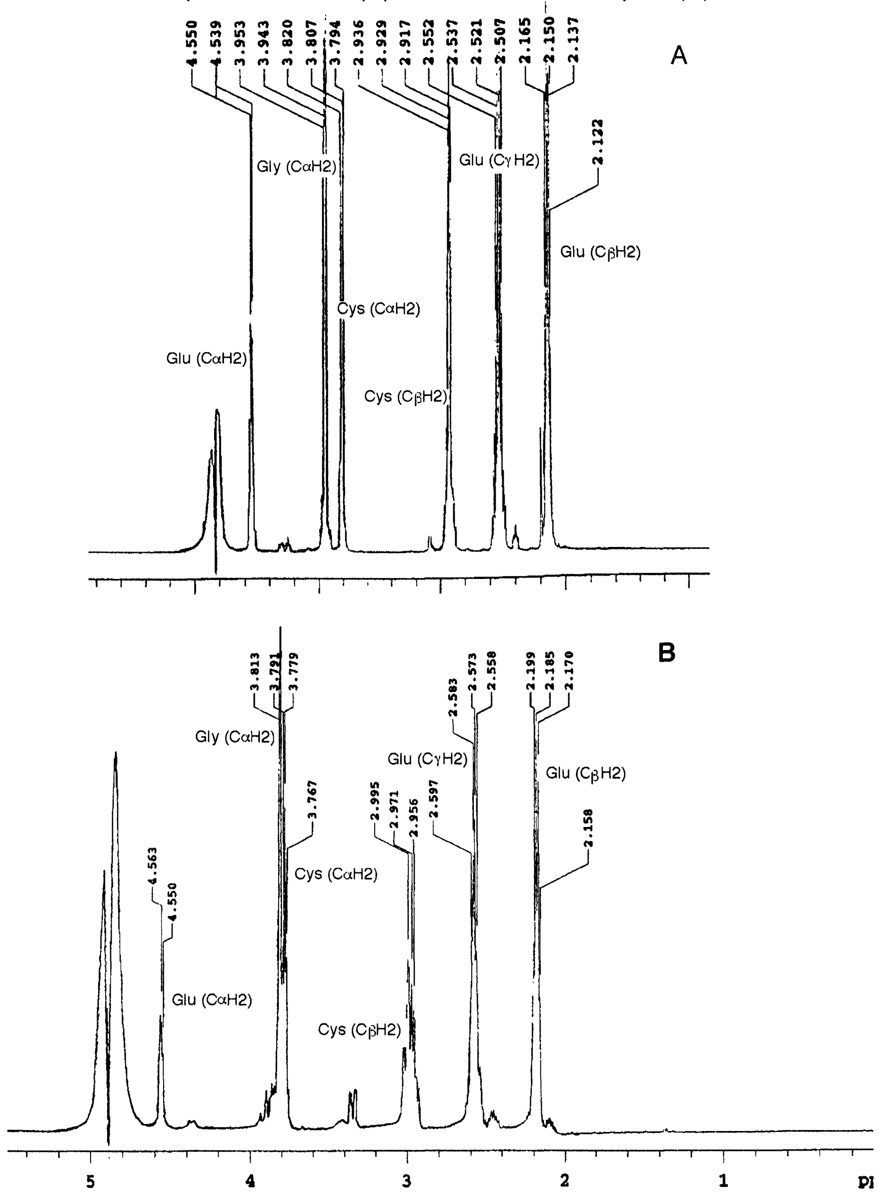

The $^{99}Tc$-GSH complex, analyzed by fast atom bombardement mass spectroscopy, showed a molecular mass between 683 Da and 726 Da. This mass corresponded to a GSH-Tc-GSH chelate complex. This complex containing a tetra or a pentavalent technetium coordinated by two thiol and nitrogen atoms of two GSH ligand and an apical oxo group (fig. 4).

HPLC radiochromatograms of the $^{99m}Tc$-GSH complex exhibit a single peak with a retention time of 12 min, the retention time of free $^{99m}TcO_4^-$ being equal to 6 min.

The radiochromatogram of the $^{99m}Tc$-GSH obtained by analysis on the biogel P2 column demonstrated that all the radioactivity was present in a single peak with an elution volume of 16 ml. Analysis of free $^{99m}TcO_4^-$ showed a single peak with an elution volume equal to 26 ml, (fig.5).

**Figure 4** : Structure of GSH-Tc-GSH complex

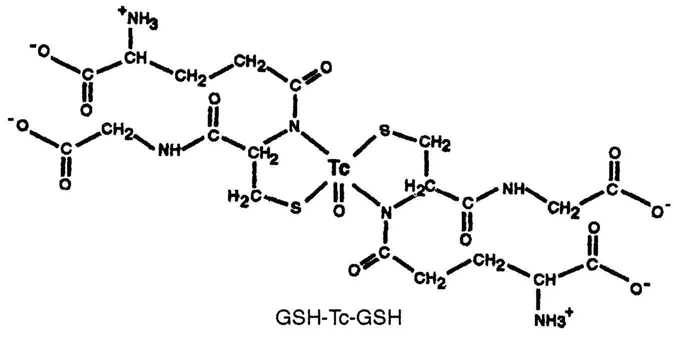

**Table III** : Biodistribution of $^{99m}$Tc-GSH complex in normal sprague dawley rats (n = 5)

| organ | %dose/g 1h | %dose/g 6h |
|---|---|---|
| kidneys | 17,5 ± 3 | 19,7 ± 3 |
| urine | 52 ± 9 | 69 ± 11 |
| blood | 5,3 ± 0,6 | 2,3 ± 0,23 |
| liver | 4,2 ± 0,5 | 2,6 ± 0,23 |
| heart | 2,5 ± 2,3 | 2,3 ± 0,23 |
| spleen | 2,17 ± 0,15 | 2,2 ± 0,15 |
| lung | 1,4 ± 0,12 | 1,1 ± 0,12 |

**Figure 5** : radiochromatogram of Tc-GSH complex obtained by biogel P2 chromatography

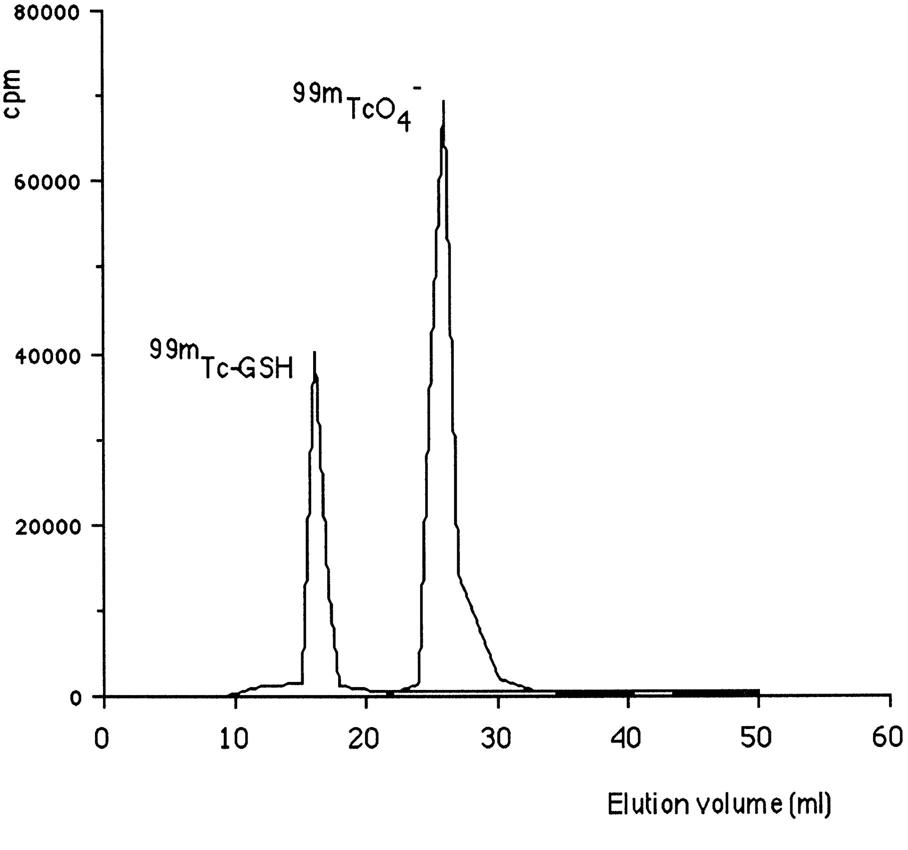

**Figure 6** : Blood clearance of $^{99m}$Tc-GSH complex in male sprague dawley rats (n = 5)

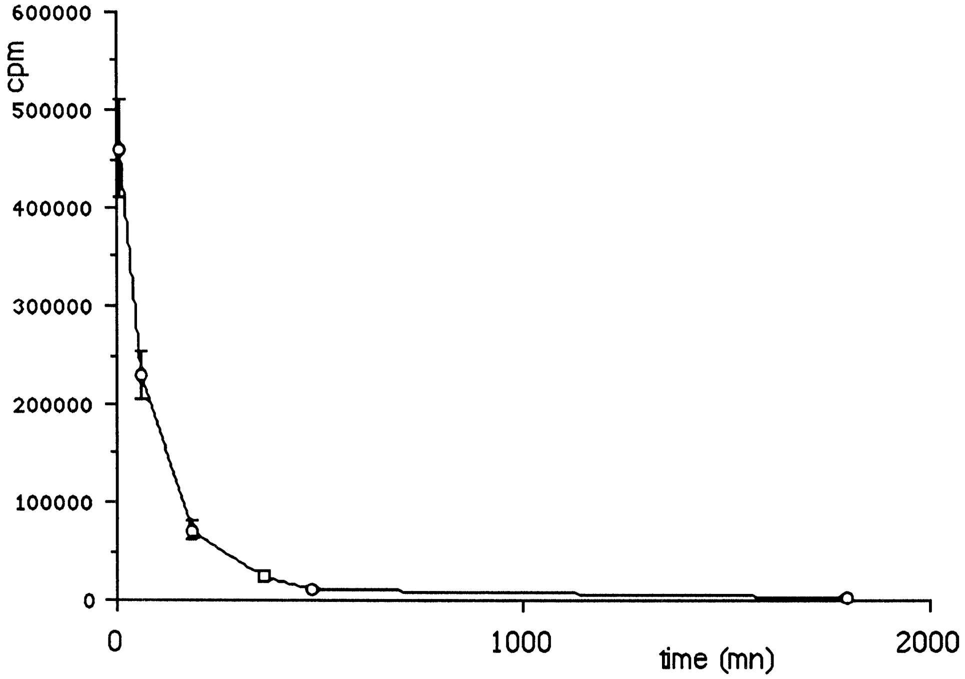

**Figure 7** : Biodistribution of $^{99m}$Tc-GSH complex in normal sprague dawley rats (n = 5)

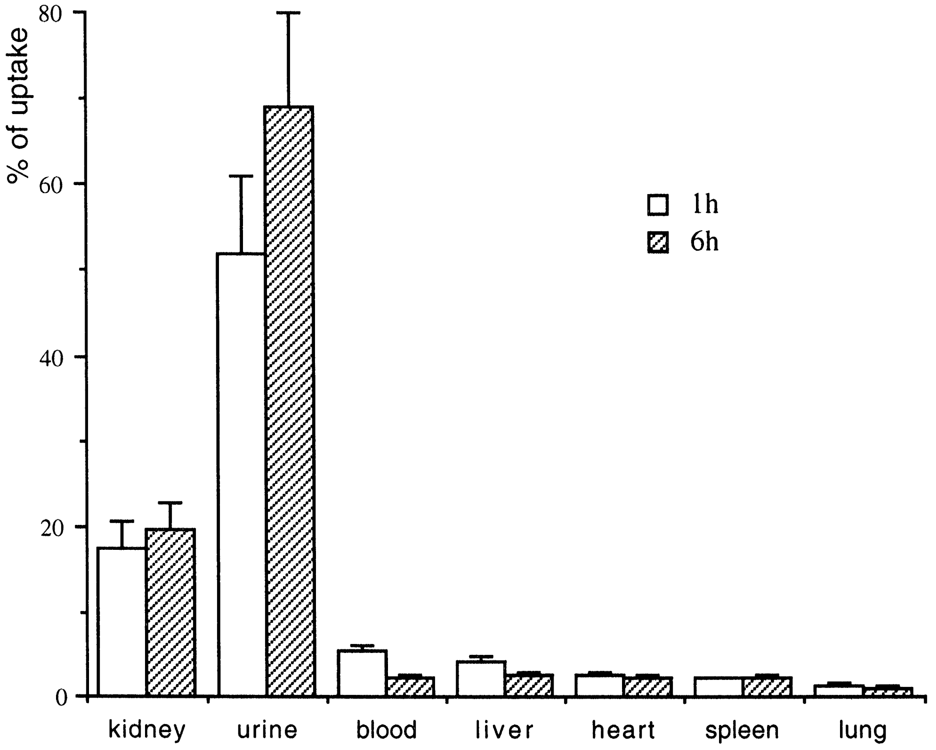

The blood clearance of $^{99m}Tc$-GSH complex in rats is shown on (fig. 6). The deconvolution of this curve demonstrated a rapid component (T1/2 (1) = 50 min, T1/2 (2) = 400 min).

The biodistribution of $^{99m}Tc$-GSH complex is given in (table III). All organs showed low uptake values except kidneys. The elimination occured via the kidneys as indicated by high radioactivity levels in urine samples (fig. 7).

### DISCUSSION

In the present study, labeling of glutathione with $^{99m}Tc$ using stanous chloride as reducing agent gave a dimer complex with a high radiochemical yield, over 95 % detected by thin layer chromatography

The NMR spectrum of the Tc-GSH complex indicated that the protons bound to cysteinyl α and ß carbons appeared to be modified, suggesting an effect of the central Tc=O core in the molecule.

Glutathione GSH could fulfill the fonction of a reducing agent and of a ligand for $^{99m}TcO_4^-$. The complexation rate seems to be higher than the reduction, since no ligand free Tc(V) compound was formed. The reduction rate diminished by decreasing the concentration of the GSH, which offers the possibility of determining the oxydation state in the complex by Sn(II) titration (20,21)

Some properties of the $^{99m}Tc$-GSH complex have already been reported (18). A highly water-soluble complex with a negative charge was formed. Blood clearance studies indicated a fast decline of radioactivity during the first hour after administration.

According to Despopoulos theory (19), an agent could be secreted through the renal tubles if it possesses anionic properties and bonding capability wich are expressed through the -CO-NH-($CH_2$)-COOH sequence.The biodistribution of the anion $^{99m}Tc$-GSH complex with the carbonyl amide sequence showed that the target organ was the kidney with a fast elimination by filtration.

Glutathione plays a critical role in the detoxification reactions by reducing $H_2O_2$ (15,24). There is increased demand for GSH in the injured and cancerous cells (23-26) and possible retention. Although the thiol group responsible for such detoxification reactions was utilized for $^{99m}Tc$ binding in the $^{99m}Tc$-GSH complex (27,28). GSH might be still be biologically active.

$^{99m}Tc$-GSH complex is a small molecule which diffuses back from inflammatory and cancerous lesions into the blood very easily (6,9,17).This intracellular concentration might be due to the transfer of $^{99m}Tc$ to another ligand inside the cell allowing concentration.(29,30).

### ACKNOWLEDGEMENTS

This work was supported by grants from the Cancer ligues dép:89 and 93. The authors thank : laboratoire de chimie, Y.Leroux, D. El Manouni and G. Leger for recording NMR spectra. F. Benazzouz for helpful experiments and G.Baillet for paper reviewing

GSH : glutathione, Tc : technetium, $SnCl_2$ : stanous chloride, cys : cysteine, gly : glycine, glu : glutamic acid, $TcO_4^-$: pertechnetate ion

### REFERENCES

1) Jones A.G. and Davison A. *Int. J. Appl. Radiat. Isot.* 1982; **33**:867-874
2) Eclman W.C., Meinken G. and Richard P. *J. Nucl. Med.* 1972; **13**:577-58
3) Tubis M., Endow J.S. *Int. J. Appl. Radiat. Isot.* 1968; **19**:835-840
4) Tubis M., Krishnamurthy G.T., Endow J.S. and Blahd W.H. *J. Nucl. Med.* 1972; **13**:652-654
5) Lin T.H., Khentigan A., Winchell H.S. *J. Nucl. Med.* 1974; **15**:34-35
6) Hagan P.I., Ayres P.R., Halpin S.E. and Chauncey D.M. *J. Nucl. Med.* 1975; **16**:531-532
7) Inoue O., Ikeda I., Kuruta K. *Radioisotopes.* 1976; **25**:23-30
8) Roizenblatt J., Buchpiguel C.A., Menguetti J.C., Caldeira J.A. and Camargo E.E. *Eur. J. Nucl. Med.* 1991; **18**:955-958
9) Ercan M.T., Aras T., Ünlenen E., Ünlü M., Ünsal I., Hasçelik Z. *Nucl. Med. Biol.* 1993; **20**:881-887
10) Ercan M.T., Bekdik C.F., Sarizi T. *Int. J. Appl. Radiol. Isot.* 1978; **29**:697-698
11) Fischman A.J., Pike M.C., Kroon D., Fucello A.C., Rexinger D., Tenkate C., Wilkinson R., Rubin R.H. and Strauss H.W. *J. Nucl. Med.* 1991; **32**:483-991
12) Mankhetrkon S., Blanchot C., Duran-Cordobes M., ElManouni D., LerouxY. And Moretti J.L. *Metal Based Drug* 1995; **2** (14):201-210
13) de Kieviet W. *J. Nucl. Med.* 1981; **22**:7.3-709
14) Hansen L, Marzilli L.G. and Taylor A. *Q. J. Nucl. Med.* 1998; **42**:280-293
15) Kaplowitz N., Aw T.Y., Ookhtens M. *Ann. Rev. Pharmacol. Toxicol.* 1985; **25**:715-744

16) Hosain F., Haddon M.J., Hosain H., Drost J. and Spencer R.P. *Nucl. Med. Biol.* 1990; **17**:151-155
17) Duman Y., Bilkay Ü., Teber E., Yüksel D., Erdem S., Argon M., Yilmaz R. and Özbal O. *Eur. J. Nucl. Med.* 1996; **19** : 1097-10
18) Ercan M.T., Aras T., Aktas T., Ünlenen E., Mocan G. and Bekdik R.P. *Nucl. Med.*, 1994, **3 3** :224-228
19) Despopoulos A. J., *J. Thero. Biol.*, 1965, **8** : 163-192
20) Person B.R.P.and Darte L. *J. Chromatogr.* 1974; **101**:315-326
21) Steigman J., Meinken G., Richards P. *Int. J. Appl. Radiat. Isot.* 1975; **26**:601-609
22) Ecklman W.C., Meinken G., Richards P. *J. Nucl. Med.* 1972; **13**:577-581
23) Bernard B.F., Krenning E.P., Breeman W.A.P., Rolleman E.J., Bakker W.H., Visser T.J., Mäkke H. and de Jong M. *J. Nucl. Med.* 1997; **38**:29-1933
24) Meister A., Anderson M.E. *Ann.Rev. Biochem.* 1983; **52**:417-423
25) Reed D.J. *Ann. Rev. Pharmacol. Toxicol.* 1990, **30**:603-631
26) Dalhoff K., Ranek L., Mantoni M. and Poulsen H.E. *Liver.*1992; **1**:341-343
27) Suess E., Malessa K., Ungersböck, Kitz P., Podreka I., Heimberger K., Hornykiewicz O. and Deecke L. *J. Nucl. Med.*, 1991; **32**:1675-1681
28) Muenze R. : *Technetium-99m radiopharmaceuticals : structure-activity relationships.* In : Deckart H, Cox PH eds. Principales of radiopharmacology. Dordrecht : Martinus Nijhoff, 1987:100-116
29) Xue L.Y., Noujaim A.A., Sykes T.R., Woo T.R. and Peng Z. *Q. J. Nucl. Med.* 1996; **40**:341-350
30) Syhre R., Seifert S., Spies H., Gupta A. and Johannsen B. *Eur. J. Nucl. Med.* 1998; **25**:793-796

**Received: June 8, 1999 - Accepted: June 25, 1999 -**
**Received in revised camera-ready format: August 23, 1999**

